# Permutation entropy in intraoperative ECoG of brain tumour patients in awake tumour surgery– a robust parameter to separate consciousness from unconsciousness

**DOI:** 10.1038/s41598-019-52949-1

**Published:** 2019-11-11

**Authors:** Nicole Lange, Sophia Schleifer, Maria Berndt, Ann-Kathrin Jörger, Arthur Wagner, Sandro M. Krieg, Denis Jordan, Martin Bretschneider, Yu-Mi Ryang, Bernhard Meyer, Jens Gempt

**Affiliations:** 1 Department of Neurosurgery, Klinikum rechts der Isar, Technical University Munich, Munich, Germany; 2 Department of Neuroradiology, Klinikum rechts der Isar, Technical University Munich, Munich, Germany; 3 Department of Anesthesiology, Klinikum rechts der Isar, Technical University Munich, Munich, Germany

**Keywords:** Adaptive clinical trial, Neurology

## Abstract

Awake craniotomies represent an essential opportunity in the case of lesions in eloquent areas. Thus, optimal surveillance of the patient during different stages of sedation, as well as the detection of seizure activity during brain surgery, remains difficult, as skin electrodes for electroencephalographic (EEG) analysis are not applicable in most cases. We assessed the applicability of ECoG to monitor different stages of sedation, as well as the influence of different patient characteristics, such as tumour volume, size, entity, and age or gender on permutation entropy (PeEn). We conducted retrospective analysis of the ECoG data of 16 patients, who underwent awake craniotomies because of left-sided brain tumours at our centre between 2014 and 2016. PeEn could be easily calculated and compared using frontal and parietal cortical electrodes. A comparison of PeEn scores showed significantly higher values in awake patients than in patients under anaesthesia (p ≤ 0.004) and significantly higher ones in the state of transition than under general anaesthesia (p = 0.023). PeEn scores in frontal and parietal leads did not differ significantly, making them both applicable for continuous surveillance during brain surgery. None of the following clinical characteristics showed significant correlation with PeEn scores: tumour volume, WHO grade, first or recurrent tumour, gender, and sex. Being 50 years or older led to significantly lower values in parietal leads but not in frontal leads. ECoG and a consecutive analysis of PeEn are feasible and suitable for the continuous surveillance of patients during awake craniotomies. Hence, the analysis is not influenced by patients’ clinical characteristics.

## Introduction

Awake craniotomies have been performed for decades to improve the extent of resection and patients’ neurological outcome in the case of lesions in eloquent areas^1,2^. It facilitates the localization and preservation of functional areas through direct electrical stimulation of the cortex through communication with and observation of the patients. Awake craniotomies can be safely performed with very low complication rates, more or less regardless of American Society of Anesthesiologists’ (ASA) classifications, body mass index, seizure frequency, tumour site or size^2–5^. Thus, optimal surveillance of the patient during different stages of sedation, as well as detecting seizure activity during surgery, remains difficult. In general anaesthesia, analysis of a real-time electroencephalographic (EEG) signal, using skin electrodes on the forehead, is widely used to continuously calculate the effects of anaesthetic drugs on the central nervous system^6^. One method of measuring an arbitrary time series based on the analysis of permutation patterns is permutation entropy (PeEn), introduced by Bandt and Pompe 2002. It is a nonparametric, nonlinear parameter that is based on Shannon’s entropy. When a time series is more chaotic, PeEn increases^7,8^.

In brain surgery, skin electrodes are not applicable in most cases. Of course, there are already several ECoG studies in the field. In general, ECoG changes during anaesthesia are similar to EEG changes; during the induction of anaesthesia, alpha waves are replaced by high-frequency beta activity. During anaesthesia, those are replaced again by low-frequency theta and delta waves^9^. But there are indeed some differences between ECoG and EEG. First of all, as placed directly on brain tissue, ECoG is not influenced by muscular activity contrary to EEG. Furthermore, it has the advantage of avoiding the scalp as a shielding effect of waves. Therefore, ECoG has already been demonstrated to be more sensitive for localizing and measuring brain lesions than scalp EEG^10^. In a study with simultaneous scalp and intracranial EEG recording, Pacia *et al*. showed that sometimes seizures can be easily identified with ECoG while there are no changes in scalp EEG^11^. In 2001, Karasawa *et al*. showed for the first time that there are wave activity patterns in the depth during deep anaesthesia which can be recorded only by ECoG, but not by skin electrodes^12^.

The first aim of this study was to analyse if previous results of PeEn in scalp EEG studies can be confirmed in ECoG. The PeEn measures information content in EEG; consequently, as there seem to be more recording of activity patterns in ECoG than in EEG during anaesthesia, it needed to be seen if PeEn scores also differ in ECoG from consciousness to unconsciousness. Furthermore, as ECoG is more sensitive in detecting localized changes in electrical brain activity, it was of interest if PeEn scores are different in recordings in different brain lobes, and if PeEn is affected immediately after stopping narcotic infusion systems. Our second aim was to identify patients’ or tumour characteristics, such as tumour volume, size, entity, age, and gender, which might influence PeEn and create pseudo-changes in consciousness where there are none.

## Materials and Methods

### Patient population

We retrospectively analysed ECoG data from our prospective database of patients who underwent awake craniotomy for resection of primary left-sided brain tumours with continuous ECoG in our centre from February 2014 to October 2016. Six patients had to be excluded due to insufficient ECoG data. We recorded patients’ and disease characteristics, including age, sex, the WHO grade of the tumour, and tumour size.

The present study was approved by the local ethics committee (Ethikkommission der Fakultät für Medizin der Technischen Universität München) and performed in accordance with the ethical standards established by the 1964 Declaration of Helsinki and its later amendments^13^ (Clinical Trial Registration Number: 284/15). Informed consent was obtained from all participants and/or their legal guardian/s.

### Anaesthetic technique

We conducted anaesthesia during awake craniotomy in asleep–awake–asleep technique. Patients underwent total intravenous anaesthesia (TIVA) with propofol and remifentanil (Remifentanil-hameln). We used laryngeal masks (Ambu® AuraGain™ Disposable Laryngeal Mask oder Ambu® AuraFlex™ Disposable Laryngeal Mask) as a device for airway management. Surgery was started in general anaesthesia. After preparation for and opening of the dura, we localized and verified the tumour via the navigation system (Brainlab). We then placed four frontal and four parietal ECoG electrodes (Inomed cortical stripe electrodes) on the cortex, and initiated continuous recording. Thereafter, we stopped infusion systems of propofol and remifentanil. We removed laryngeal masks after patients were awake and opened their eyes on command. Then we conducted tumour resection while patients spoke continuously with a neuropsychologist. Meanwhile, we stopped the EEG recording after a defined time period of wakefulness. We put patients back into general anaesthesia for the rest of the surgical procedure after the resection of critical tumour regions.

### EEG analysis and processing

One method of measuring an arbitrary time series based on analysis of permutation patterns is PeEn, which was introduced by Bandt and Pompe in 2002^7,8^.

We extracted several defined sequences of the continuously recorded ECoG within the time point of interest, and we calculated PeEn. For analysis, we cut each segment into six segments of t = 10 s, and calculated PeEn for each of the six segments in the four frontal and four parietal leads. At every time point, we assessed the means of frontal and parietal leads.

**Figure 1:**
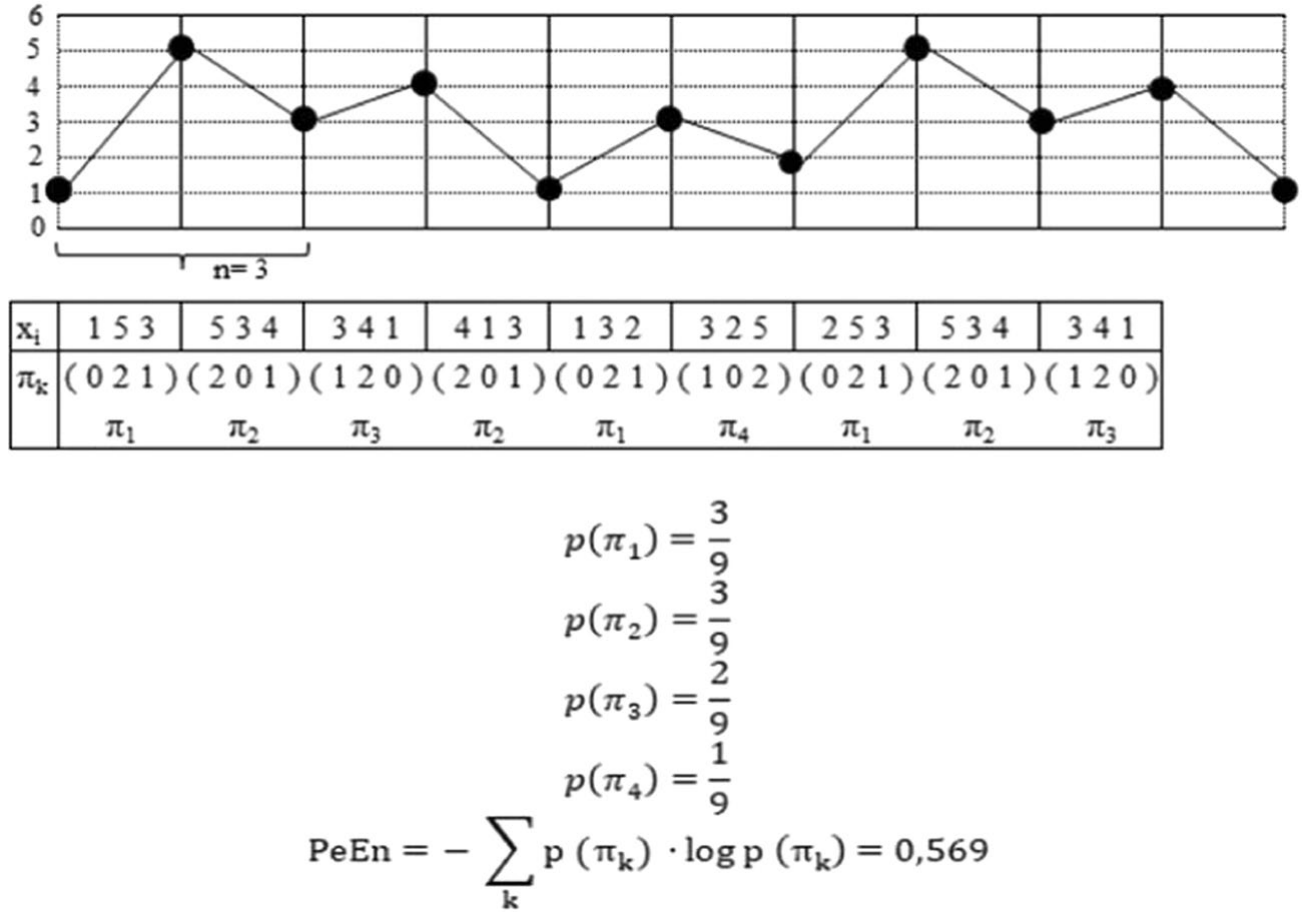
Exemplary time series with a calculation scheme for the parameter permutation entropy (PeEn) on the basis of an embedding dimension *n* = 3. The probability *P*(π_*k*_) for the occurrence of every obtained permutation π_*k*_ is calculated, thus defining a probability distribution of the permutations. PeEn is obtained as the Shannon entropy of the resulting probability distribution of π_*k*_. It quantifies the amount of different amplitude rankings π_*k*_ of length *n*. For this study, the following settings for the calculation of PeEn were used: dimension n = 5, order = 3, τ = 5 and sampling frequency fs = 200 Hz^14^.

Figure 1 shows an exemplary time series with a calculation scheme for the parameter PeEn. As seen in the figure, for the calculation of PeEn, the dimension *n* must be determined, which describes how many amplitude values are included in one permutation (recommended as a dimension between 3 and 7)^7^. Then we placed the *n* amplitude values in order of their value (e.g. the n = 3 values in Fig. 1: 1, 5 and 3 receive the rank 0-2-1; they form the permutation π_k_ = 0-2-1). The next permutation is defined after lag τ = 5. Based on Shannon’s entropy, PeEn is calculated by the probability distribution of all types of permutations π_k_.

The formula for PeEn is,

$${\rm{PeEn}}=-\,\sum _{{\rm{k}}}{\rm{p}}({{\rm{\pi }}}_{{\rm{k}}})\cdot \,\log \,{\rm{p}}({{\rm{\pi }}}_{{\rm{k}}})$$

If there is just one type of permutation, the value of PeEn is 0. If all permutations occur equally frequently, PeEn achieves the maximum value (PeEn_max_ = log(k)^8,14^.

For this study, we used the following settings for the calculation of PeEn:

dimension n = 5, time lag τ = 5, sampling frequency fs = 200 Hz.

**Figure 2:**
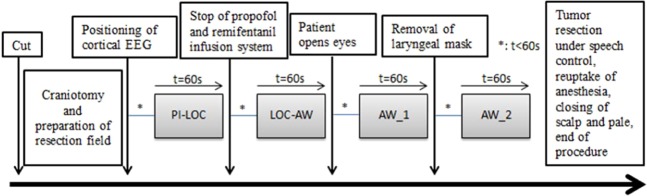
Flow chart showing different time points of EEG measurement during one awake surgery. PI-LOC = propofol-induced loss of consciousness; LOC-AW = state of transition from loss of consciousness to wakefulness; AW_1 = awake 1; and AW_2 = awake 2.

Figure 2 is a flow chart showing the different time points of ECoG measurement during an exemplary awake surgery. We extracted four segments of the record of each patient with t = 60 s at the following time points to calculate PeEn:

1 = propofol-induced loss of consciousness (PI-LOC): Baseline ECoG after placing electrodes in propofol-anaesthesia

2 = LOC-AW: shortly after stopping propofol and opioid perfusors, representing the state of transition from loss of consciousness to wakefulness.

3 = awake 1 (AW_1): shortly after patient opened his eyes for the first time either to a vocal command or spontaneously.

4 = awake 2 (AW_2): shortly after removal of laryngeal mask (after patient has responded to commands).

For the whole ECoG processing—from filtering to the calculation of parameters—we used LabView 8.5 (National Instruments, Austin, eTX, USA).

Usually, frequencies exceeding 30 Hz in EEGs are filtered because frequencies above this cut-off may be caused by muscular activity^15^. PeEn is robust against artefacts caused by muscular activity because it is not calculated with absolute values^14^. Nevertheless, we applied a bandpass-filter between 0.5 Hz and 30 Hz (Butterworth filter, order 3) to sustain similar conditions to previous studies with scalp analyses.

### Imaging

The tumour volume was assessed via fluid-attenuated inversion recovery (FLAIR) or T1-weighted contrast-enhanced magnet resonance imaging (MRI) sequences using semiautomatic segmentation software (ITK-SNAP, www.itksnap.org)^16^.

### Statistical data analysis

We used IBM SPSS Statistics version 22.0 (IBM Corporation, New York, USA) and GraphPad Prism version 7.04 for Windows (Graphpad Software Inc., La Jolla California, USA) for statistical analysis. For the whole analysis, nonparametric tests were used due to the small sample size. For comparison of PeEn at different levels of patients’ wakefulness, the Friedman’s test and Wilcoxon signed-rank test were applied. To compare data of frontal and parietal electrodes, the Wilcoxon signed-rank test was used. We evaluated correlation for tumour volume, patient’s age, tumour entity, and tumour recurrence by using correlation tests, the Mann–Whitney U test and the Kruskal–Wallis test.

## Results

### Clinical characteristics of the patient collective

We analysed the EcoG data of 22 patients who underwent awake craniotomy and resection of left-sided primary brain tumour between February 2014 and October 2016 in our centre (clinical characteristics see Table 1). In 16 patients (10 male, 6 female), ECoG data were registered for all time points, and the patients were put into general anaesthesia for closure so that they could be included in this study. The mean age was 42 (range of 24–66 years).

**Table 1:** Demographic table of patients’ and tumour characteristics.

Patients’ and tumour characteristics	
age	Mean: 42 years, SD: 13 years, min.: 24 years, max.: 66 years
gender	10 male/6 female
tumour volume	Mean: 51.2 cm^3^, SD: 41.4 cm^3^, min.: 1.9 cm^3^, max.: 130.0 cm^3^
WHO grade	6 grade II/5 grade III/5 grade IV tumours
localization	7 frontal/6 temporal/1 frontotemporal/1 frontoparietal/1 temporoparietal
entity	8 astrocytoma/4 glioblastoma/3 oligodendroglioma/1 oligoastrozytoma
initial or recurrent tumour	7 initial/9 recurrent tumour

The mean tumour volume was 51.2 cm^3^ (range 1.9–130 cm^3^). Seven patients had initial diagnosis, and nine had a recurrent brain tumour. The tumour was located in the left frontal lobe in 43.8% of cases, in the temporal lobe in 37.5% of cases, and in the frontal and temporal lobe, frontal and parietal lobe, and temporal and parietal lobe in 6.3% of cases. There were consistent lesions in the speech areas of the brain, including frontal opercular, insular, and temporal superior gyrus and the adjacent parietal parts, indicating awake tumour surgery to evaluate patients’ speech performances. Six tumours were classified as World Health Organization (WHO) grade II (37.5%), and five were classified as WHO grades III and IV (31.3%). Tumour entities were as follows: eight astrocytomas, five glioblastomas, two oligodendrogliomas, and one oligoastrocytoma. (See Table 1).

Thirteen patients (81%) reported at least one epileptic seizure prior to surgery, and four of them reported multiple seizures. Antiepileptic medication (levetiracetame in most cases, as well as vimpat, lamotrigine, and lacosamide in two cases) was taken in those four cases at the time of admission (and perioperatively). In the remaining nine patients, it was prescribed postoperatively for the following six months.

Symptoms leading to diagnosis, apart from seizures, included aphasia in five patients (31%), headache and concentration disorders in four patients (25%), and pareses in two patients (13%).

Postoperatively, transient aggravation of symptoms or new transient paresis were recorded in six patients (38%). There were no new permanent deficits.

### PeEn in frontal and parietal leads

**Figure 3:**
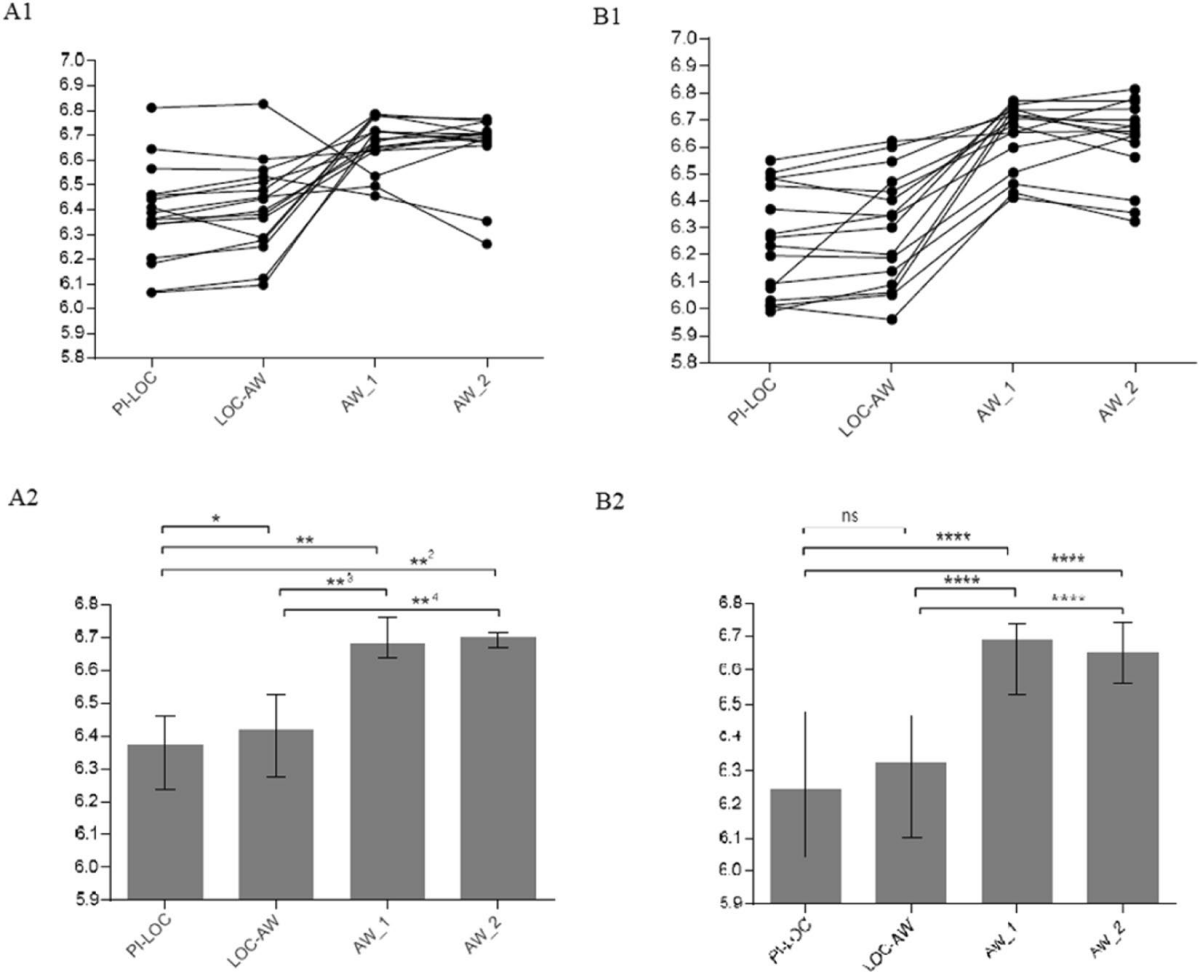
Comparison of PeEn scores in frontal and parietal leads at different time points. 4 frontal leads (means) and 4 parietal leads (means) are shown. (**A1**) course of PeEn scores in frontal leads at different time points for 16 patients. (**B1**) of PeEn scores in parietal leads at different time points for 16 patients. (**A2**) Wilcoxon analysis of frontal leads at different time points (median and interquartile ranges). *p = 0.021, **p = 0.002, **^2^p = 0.002, **^3^p = 0.002, **^4^p = 0.004. (**B2**) Wilcoxon analysis of parietal leads at different time points (median and interquartile ranges). ****p < 0.001, ns = not significant.

We calculated PeEn at PI-LOC, LOC-AW, and AW_2 for 16 patients in both frontal and parietal leads. We calculated AW_1 for 15 patients (one is missing due to the missing time point). Medians in frontal leads were as follows: PI-LOC: 6.37; LOC-AW: 6.42; AW_1: 6.67; and AW_2: 6.7. We conducted the Friedman test and the Wilcoxon signed-rank test to further evaluate PeEn score differences at the defined time points (Table 2). We could show that PeEn scores are significantly higher in awake patients than in patients under anaesthesia (p ≤ 0.004) and are significantly higher in the state of transition than under general anaesthesia (p = 0.023). (See Fig. 3).

**Table 2:** Test statistics with z-value and p-value of the Wilcoxon signed-rank test for PeEn of each pair of time points in frontal and parietal leads.

	Frontal leads			Parietal leads		
	Median of differences	Z-value	p-value	Median of differences	Z-value	p-value
PI-LOC/LOC-AW	0.04	2.276	0.023	0.04	1.81	0.07
PI-LOC/AW_1	0.31	2.897	0.004	0.37	3.408	0.001
PI-LOC/AW_2	0.31	3.051	0.002	0.33	3.516	<0.001
LOC-AW/AW_1	0.27	2.726	0.006	0.32	3.408	0.001
LOC-AW/AW_2	0.28	2.896	0.004	0.32	3.516	<0.001
AW_1/AW_2	−0.01	0	1	0	0.398	0.691

A statistical analysis of parietal leads showed similar results (medians: PI-LOC: 6.25, LOC-AW: 6.32, AW_1: 6.68 and AW_2: 6.67). Although there was no significant difference between the state of transition and complete anaesthesia (p = 0.07), scores in awake patients are significantly higher than in patients under anaesthesia (p ≤ 0.001).

Paired comparison of frontal and parietal leads did not show significantly different scores neither at wakefulness nor during anaesthesia (PI-LOC: MD = 0.02, p = 0.27; LOC-AW: MD = 0.07; p = 0.18; AW_1: MD = 0.01, p = 0.82; AW_2: MD = 0.02, p = 0.49). Figure 3 shows a comparison of PeEn scores in frontal and parietal leads at different time points, as well as the course of median PeEn values.

### PeEn and variables

We also analysed correlations between PeEn and patients’ clinical characteristics, such as tumour volume, tumour entity, WHO grades, age, and gender. Due to the small sample size, we conducted correlation analyses instead of multiple regressions.

First, we correlated PeEn with tumour volume. Spearman’s coefficient did not reach significance levels, neither for the analysis of the whole collective (FLAIR sequences) nor for the subgroup of seven contrast-enhancing tumours (T1-weighted contrast-enhanced sequences).

We also conducted analysis of the influences of tumour entity and WHO grades on PeEn. To avoid multiple testing, we only used data of two time points (PI-LOC: patient under anaesthesia, AW_2: patient awake) to compare the different groups. Neither could the Mann–Whitney U test find any significant differences between PeEn scores in patients with initial and recurrent tumours, nor could the Kruskal–Wallis test for patients with tumours of different WHO grades.

Concerning patient characteristics, we evaluated differences between age and sex. Scores of PeEn between male and female patients did not differ significantly in the exact Mann–Whitney U test. The correlation analysis of age and PeEn scores did not indicate significant correlations between the two variables, but grouped analysis, conducting an exact Mann–Whitney U test comparing patients older than 50 years (n = 5) and patients younger than 50 (n = 11), revealed significantly higher scores of PeEn between the state of anaesthesia and wakefulness for younger patients in parietal leads than for older patients (PI-LOC: p = 0.04, AW_2: p = 0.005). Scores between age groups were similar in frontal leads (PI-LOC: p = 0.91; AW_2: p = 0.44).

## Discussion

Awake craniotomies are an essential tool to improve the extent of resection and patients’ neurological outcome in the case of lesions in speech-eloquent areas. In this analysis of 16 patients who underwent awake craniotomies with lesions in speech-eloquent areas in our centre between 2014 and 2016, we verified the applicability of ECoG and consecutive analysis of PeEn with a special focus on the influence of patients’ and tumour-specific characteristics.

The use of EEG for intraoperative surveillance remains controversial^17,18^. A lot of narcotic monitors have come up to help separate consciousness from unconsciousness to avoid intraoperative awareness, such as the *Bispectral Index (BIS)* (Covidien, Mansfield, MA, USA), the *Entropy Module* with integrated spectral entropy (GE Healthcare, Helsinki, Finland), and the *Narcotrend* (MMH, Hannover, Germany). Especially for the BIS monitor, there are various studies that show the benefit in terms of reducing anaesthetic drug use and avoiding intraoperative awareness^17,18^. Thus, clinical benefit of all those monitors is still discussed controversially^19–22^. PeEn, which has not yet been implemented in a narcotic monitor, is a highly interesting approach for a new monitoring system. Previous studies with scalp EEG have shown that PeEn is robust against episodes of high frequency (e.g. caused by signal artefacts or muscular activity) and is therefore suitable for intraoperative measurements^7,23^. Furthermore, it has shown to yield a classification accuracy of over 90% to distinguish between awake and sleeping phases during general anaesthesia, regardless of the substances used^24^. It has been evaluated for use in children and adults, and is therefore a widely used tool for the assessment of anaesthetic depth^8,25–27^. Jordan *et al*.^14,28^ showed significantly lower values of PeEn in patients under anaesthesia than in awake patients in frontal leads, but not in parietal or temporal leads. In another study in 2013, they demonstrated that PeEn in frontal leads was able to separate consciousness from unconsciousness, whereas power spectral analysis only showed weak effects at different anaesthetic levels^29^. A study of PeEn during sevoflurane narcosis published by Ranft *et al*. showed a reduction in PeEn in all frontal, temporal, parietal, and occipital leads^30^. The PeEn scores were not influenced in any way by the localization on different brain lobes. Thus, the decrease in PeEn values in frontal and parietal electrodes means reduced information content. To date, there has been no study of PeEn in ECoG to make the parameter applicable for intracranial neurosurgery.

In our study, PeEn could be easily calculated and compared using frontal and parietal cortical electrodes. The comparison of cortical PeEn scores during propofol-induced anaesthesia, the state of transition from loss of consciousness to wakefulness, and the awake phases of the surgery showed significantly different values in the frontal leads (p = 0.001) and the parietal leads (p < 0.001), confirming previous results of skin-electrodes. Regarding parietal leads, the Wilcoxon signed-rank test revealed significant differences between all phases of consciousness, except the transient phase and patients under anaesthesia. This may also be contributed to the study design with just one sample shortly after stopping propofol- and remifentanil-infusion systems. As this study was the first evaluation of PeEn in electrocorticography, the following detailed analysis of PeEn during the transient phase in cortical electroencephalograms with several time samples considering the half-life of propofol should be assessed. Interestingly, in two patients PeEn goes down in the frontal leads but goes up in the parietal. Retrospectively, no disturbance in recording or epileptic seizure was identified at this time point. So finally, the reason remains unclear.

PeEn scores in frontal and parietal leads did not differ significantly at any time point, making them both applicable for continuous surveillance during brain surgery.

Our results—showing PeEn in ECoG to be able to separate consciousness from unconsciousness independent of electrode localization—confirm for the first time previous results in scalp EEG.

Analysing the influence of other variables on PeEn scores, which could disturb their interpretation, we conducted correlations between PeEn scores and the following clinical characteristics: tumour volume, WHO grade, initial or recurrent tumour, and gender. Considering previous research about tumours and changes in EEG patterns, we expected PeEn scores to differ depending on tumour size and entity. Previous studies have shown changes in the EEG induced by tumours. Yet, earlier, shortly after the invention of EEG, the so-called polymorphic delta waves were identified at structural brain lesions^31,32^. Those waves seem to be correlated with tumour size^33^. Moreover, different EEG patterns seem to occur in high-grade glioma compared to low-grade glioma. Less malignant tumours induce localized EEG changes, mainly in theta waves, followed by delta wave changes. Glioblastomas seem to have more diffuse impact on EEG; even the ground rhythm of EEG was impaired in those lesions^32,34,35^.

Therefore, we wanted to see if and how PeEn scores were influenced by these tumour characteristics as well as by patients’ characteristics. Surprisingly, none of the tumour characteristics showed significant correlations, neither during general anaesthesia nor when the patient was awake (p > 0.05 for all analysis). Patients’ age was the only factor playing a significant role in the PeEn values, thereby showing significantly higher values in parietal leads for patients younger than 50 years (PI-LOC: p = 0.04, AW_2: p = 0.005). This difference could not be shown in frontal leads.

This age-related asynchrony in EEG is well known and has been shown in many previous studies^36–38^. It is explained by an age-related reduction of the cortical connectivity, resulting in a smaller amplitude of oscillations and thereby lower PeEn values.

## Conclusion

In summary, our findings show for the first time that PeEn in ECoG during awake craniotomy is a feasible and suitable option for the surveillance of patients’ consciousness during the entire surgical procedure. Thus, it is not influenced by patient- or tumour-specific characteristics and shows the same age-related changes as previous EEG measurements.
